# Cost-effective scat-detection dogs: unleashing a powerful new tool for international mammalian conservation biology

**DOI:** 10.1038/srep34758

**Published:** 2016-10-10

**Authors:** Joseph D. Orkin, Yuming Yang, Chunyan Yang, Douglas W. Yu, Xuelong Jiang

**Affiliations:** 1 State Key Laboratory of Genetic Resources and Evolution, Kunming Institute of Zoology, Chinese Academy of Sciences, 32 Jiaochang Donglu, Kunming, Yunnan 650223, China; 2 Department of Anthropology, Washington University in St. Louis, 1 Brookings Drive, St. Louis, MO 63130, USA; 3 Department of Anthropology and Archaeology, University of Calgary, 2500 University Drive N.W., Calgary, Alberta, T2N 1N4, Canada; 4 Kunming Police Dog Training Base, Chinese Ministry of Security, 579 Baiyunlu Kunming, Yunnan, 650204, China; 5 School of Biological Sciences, University of East Anglia, Norwich Research Park, Norwich, Norfolk NR47TJ, UK

## Abstract

Recently, detection dogs have been utilized to collect fecal samples from cryptic and rare mammals. Despite the great promise of this technique for conservation biology, its broader application has been limited by the high cost (tens to hundreds of thousands of dollars) and logistical challenges of employing a scat-detection dog team while conducting international, collaborative research. Through an international collaboration of primatologists and the Chinese Ministry of Public Security, we trained and used a detection dog to find scat from three species of unhabituated, free-ranging primates, for less than $3,000. We collected 137 non-human primate fecal samples that we confirmed by sequencing taxonomically informative genetic markers. Our detection dog team had a 92% accuracy rate, significantly outperforming our human-only team. Our results demonstrate that detection dogs can locate fecal samples from unhabituated primates with variable diets, locomotion, and grouping patterns, despite challenging field conditions. We provide a model for in-country training, while also building local capacity for conservation and genetic monitoring. Unlike previous efforts, our approach will allow for the wide adoption of scat-detection dogs in international conservation biology.

Collecting a large number of biological samples from free-ranging animals remains the overarching limitation for mammalian conservation genetics and molecular ecology. While great advances have been made in recent years, the focus has been on how to do more with less: deeper sequencing and fewer samples^1^,^2^. This is nowhere more apparent than in primate biology, which often requires obtaining DNA from arboreal, small, fast, cryptic, and nocturnal animals with large home ranges. Combined with the facts that most primates are threatened, and that almost all of them are distributed throughout the Global South—often in difficult to reach jungles and mountainous terrain—international population and conservation genetic projects are routinely deemed impossible before they can begin. For most populations of most species, the cost per sample is simply too high and too time consuming. As a result, the trajectory of decades of genetic research has been to deepen the study of a few, well-known populations, and to write-off the majority of this diverse radiation as unknowable.

One possible solution to this problem could be found with the emergence of scat-detection dogs used for wildlife tracking; however, no model exists for the use of them in the Global South that is practical for both local conservation stewardship and long-term international scientific collaboration. While dog teams have found hundreds of samples from rare mammals that are unhabituated to human observers^3^,^4^,^5^,^6^,^7^, the expansion of this approach remains limited by its almost singular dependence on three detection dog training groups based in the United States: (1) Working Dogs for Conservation; (2) Pack Leader; and (3) The University of Washington Center for Conservation Biology. While the effectiveness and pioneering importance of these groups cannot be overstated, the global application of detection dogs to ecology and conservation remains limited by the availability and high price of working with an established detection dog team. For example, the only such study of primates—a two-month effort to locate scat samples from cross-river gorillas at two sites in Cameroon—cost $98,000^7^. Unfortunately, such high costs are typical. Long *et al.*^8^ estimated that employing a detection dog group for a several-month-long, single-dog survey in the United States would add ten to twenty thousand dollars to preexisting research costs. Recently, an Australian team independently trained a detection dog for koalas^9^, but this approach is not generalizable, because of the high cost of international animal transportation and quarantine—potentially tens of thousands of dollars alone. As such, it is simply not reasonable to expect that most conservation biologists and molecular ecologists can employ foreign dog teams.

The prospect of collaborating with local police in habitat countries to train dogs to locate scat offers a tremendous opportunity to expand the range of both conservation genetics and molecular ecology. Police in most countries use dogs for detection operations, and we suggest that collaborating with them could allow for the use of scat-detection dogs in a cost-effective manner. First, doing so would eliminate the cost of international travel and handler fees if a project member or local conservationist were to manage the dog. Second, because of the scale of most police dog operations, the cost of dog and handler training can be dramatically lowered.

We contend that sympatric primates allow for an ideal test case for an international collaboration employing a police trained scat-detection dog. Primates are broadly distributed throughout the Global South: half of all species are threatened with extinction, and almost all individuals are unhabituated to human observers. Furthermore, there is an ongoing need for improved techniques to locate and identify scat from free-ranging primates, and hiring a detection dog team is cost-prohibitive in all but the most well-funded cases^7^. Additionally, primates consume a wide range flora (fruit and/or leaves) and fauna (insects and small vertebrates), which could affect detection dog accuracy. Given that almost all published accounts of scat-detection dogs examined Carnivora or terrestrial mammals^3^,^4^,^5^,^7^,^8^,^10^,^11^,^12^,^13^,^14^,^15^,^16^, using a detection dog to search for scat from multiple sympatric primates will allow for a broad, yet stringent test of our approach.

Here, we report an international collaboration between Washington University in St. Louis, The Chinese Academy of Sciences, and the Chinese Ministry of Public Security to test the effectiveness of a locally trained police detection dog to locate feces for genetic identification from multiple species of endangered, ecologically diverse, unhabituated primates. First, we trained a dog (a Belgian Malinois named, “Pinkerton”) to identify scat from western black crested gibbons (*Nomascus concolor*), Indochinese gray langurs (*Trachypithecus crepusculus*), and stump-tailed macaques (*Macaca arctoides*) (Fig. 1). Second, we sampled 12 sites across two mountain ranges in Yunnan, China (Wuliangshan and Yongde Daxueshan), where surveys indicate or local people believe these primates range (Fig. 2). Finally, we sequenced taxonomically informative genetic markers from the collected samples to validate their source species identity and the accuracy of both our detection dog team and human-only teams. By uniting Chinese and American academic field biologists with local Chinese police dog trainers, we demonstrate how scat-detection dogs can be used for conservation and ecology throughout the Global South at low cost.

## Results

In total, we collected 202 putative non-human primate fecal samples from 12 sites in two mountain ranges: nine sites in Wuliangshan and three sites in Yongde Daxueshan. Of the 202 putative samples, we identified taxonomically informative mtDNA markers (primate family-specific d-loop or vertebrate-specific CO1) from 157 (~78%) of them. No PCR product could be amplified from the remaining 45 samples. 137 of these sequences aligned unambiguously to *Nomascus* (71), *Trachypithecus* (21), or *Macaca* (45) using NCBI nBLAST searches (Table 1). Our d-loop primers amplified successfully in 135 of these cases, and two additional *Nomascus* samples were identified with the vertebrate-specific CO1 mini-barcode primers. 20 samples aligned to non-target species after amplification with the CO1 mini-barcode primers: five *Homo sapiens* and 15 galliform birds. 31 samples were collected from Yongde Daxueshan, 14 of which yielded primate DNA, and the remaining 171 samples were collected from Wuliangshan, 123 of which yielded primate DNA.

Given the rarity of sequence data from *N. concolor* and *T. crepusculus* in the NCBI database, we were unable to identify the species level designation of each sample. However, because these species are the only members of the genera *Nomascus* and *Trachypithecus* inhabiting our field sites, we are confident that all gibbon and langur samples are from our target species. Sequences from 31 samples aligned closely (<2% sequence distance) with Yunnan isolates of *M. arctoides*; however, we were unable to confidently identify the *Macaca* species from 14 samples, which has sequence identity closest to *M. assamensis* (5–6% sequence difference; 6–8% difference from known Yunnan isolates). This is not surprising given that multiple *Macaca* species are present in both the Wuliang and Yongde Daxueshan Mountains, not all of which have been widely sampled at this locus.

The human-dog team collected 127 of the 202 putative non-human primate samples, and the human-only team collected an additional 75 without identification by the dog. Of the samples that were collected by the human-only team, 34 (45%) were correct, 11 (15%) were incorrect, and 30 (40%) were unidentifiable. In contrast, the human-dog team correctly identified 103 (81%) samples, made nine (7%) errors, and collected 15 (12%) samples that could not be identified (Tables 1 and 2). We define “unidentifiable” samples as those from which we could not amplify any informative locus and “incorrect” samples as those which amplified a sequence from a non-target vertebrate. Thus, excluding/including the unidentifiable samples, the accuracy rates are 76%/45% and 92%/81% for the human-only and human-dog teams, respectively. Additionally, there was a noticeable increase in the dog team’s success rate between the first and second field seasons (Fig. 3).

We built mixed effect linear models to determine the effectiveness of the dog-human team against the human-only team (Table 3). To accommodate for the hierarchical structure of our data, we included a nested random effect (sites within mountains) in each model. Team membership was found to significantly predict the number of correct DNA samples collected (Table 3: Model 1); the number of correct DNA samples collected when accounting for unidentifiable samples (Model 2); the number of unidentifiable samples (Model 4); and the number of useless samples (i.e. those which were from non-target taxa or unidentifiable (Model 5). Team membership did not significantly predict the number of samples collected from non-target taxa when accounting for the number of samples collected (Model 3).

## Discussion

We have shown that it is possible to overcome both the logistical challenge and high cost of international fieldwork with a scat-detection dog by collaborating with local police trainers to collect a large number of fecal DNA samples from multiple species of endangered, unhabituated, arboreal primates. For the first time, we have used a scat-detection dog to locate and identify fecal samples from multiple sympatric primates. Our results demonstrate that the regular use of scat-detection dogs by conservation biologists should be expanded to include animals with broader dietary and ecological conditions. Our dog team made few errors, and it is likely that most of these failures resulted from the degraded DNA found in old scats rather than from incorrect identification by the dog. These results are consistent with the success rates that have been found by scat-detection dog teams working with different species, and they demonstrate that scat-detection dogs can and should have a broader role in both molecular ecology and conservation biology.

Fecal genetic data have broad utility for informing both conservation biology and broader research applications^17^,^18^. Fecal DNA can be used for non-invasive population monitoring^14^,^19^, assessments of population viability and inbreeding^20^,^21^, identifying rates of dispersal and gene flow between forest fragments^22^,^23^, studies of gut microbiota^24^,^25^, genomics^2^,^26^, as well as phylogeography and evolutionary genetics^27^,^28^. Genetic results can be used to inform conservation managers about where to construct forest corridors, which animals to translocate, how to maintain population-wide genetic diversity, and which habitat regions to prioritize for protection. For example, we used the samples collected in this study to identify landscape effects on population structure within *N. concolor* in Yunnan^29^. Not only is this of relevance to the field of molecular ecology, but it also will allow us to lobby for the designation of a new provincial nature reserve in currently unprotected forest inhabited by multiple primate species. We contend that the broader application of scat-detection dogs could have a transformative effect on both conservation biology and molecular ecology.

We also suggest that local zoos can be excellent sources for training scats for detection dogs, depending on the particularity of research questions, and the degree of sympatry. We were unable to obtain *N. concolor* training scats for Pinkerton, so we used scat from a congeneric non-sympatric gibbon, *N. leucogenys*, instead. Likewise, were only able to acquire training scats from *M. arctoides*, and Pinkerton’s ability to find scat from a second unidentified macaque species was a positive development. By amplifying taxonomically informative genetic markers from each collected fecal sample, we were able to confirm target species using a detection dog that was trained broadly with zoo-collected feces.

We have also demonstrated that detection dogs can locate scat from unhabituated arboreal primates in extreme terrain. Arboreal primates often defecate from elevations of 10–30 meters above ground, causing much of a fecal bolus to be caught in and fragmented by tree limbs, leaves, and branches. The large number of scats we collected from gibbons, which are near obligate arborealists, demonstrates that these challenges do not preclude the use of a detection dog team for arboreal primates.

Our results suggest that the diet of a target species is not likely to have a pronounced effect on the ability of a detection dog to find scat. Given the preponderance of data in the literature on detecting Carnivoran scat^3^,^4^,^5^,^10^, it has been unclear how reliably a detection dog could identify target scat produced by animals consuming a strictly (or overwhelmingly) plant-based diet. Although two recent studies have successfully collected scat from gorillas^7^ and koalas^9^, other attempts to collect scat from herbivores^15^,^16^ have faced challenges from low population densities. The diets of the three primate families studied here are overwhelmingly plant-based, but differ substantially in preferred foods and manner of digestion (e.g. langurs are foregut fermenters with multi-chambered stomachs)^30^,^31^,^32^,^33^. The high rate of successful identifications from multiple primate species suggests that other confounding effects, such as the local abundance of a target species, could explain the different levels of success encountered by some dog teams.

In both absolute and relative terms, the detection dog team had higher success rates than did the human-only searchers (Tables 1 and 2). Our mixed effect linear models indicate that the human-dog team significantly outperformed the human-only team by locating more correct samples (irrespective of whether they contained amplifiable DNA), fewer unidentifiable samples, and fewer useless samples (Table 3). These relationships are evident across sites and mountains. While the human-dog team collected more correct samples than did the human-only team, the most striking observation is the three-fold difference in the number of unidentifiable samples collected by the respective teams (12% and 40%, respectively). We suggest that this difference in error rates is attributable to the different manners in which humans and dogs identify scat. Both teams collected fecal samples irrespective of their age and condition, based upon visual cues or when Pinkerton indicated that samples were of target-species origin. It is more likely that old, dry, and discolored scats will appear ambiguous to the human eye than to the olfaction of a dog. In addition to the substantially higher successful extraction rate for the human-dog team, Pinkerton was consistently able to identify training scats that had been desiccated for more than one year, suggesting that a dog, unlike a human, can reliably identify primate scat irrespective of its age and condition. The use of a scat-detection dog buffers the risk that collection of old samples will increase the time and cost of failed lab work. Although some of our older samples yielded discolored DNA that did not PCR amplify despite multiple extraction techniques, many were successful for mitochondrial and microsatellite analyses^29^, suggesting that researchers need not reflexively avoid older samples.

It is important to reiterate that our comparison between the dog team and the human-only team is an opportunistic one, given the constraints and realities of working with endangered primates in China. We were required to have new assistants at each site (often rotating individuals) and could not always find multiple people to employ due to seasonal farming responsibilities. It is possible that a continuous, experienced human-only team would have been more successful, but in order to safely navigate the new terrain at each site, it was imperative to work with local assistants. Nonetheless, our results are consistent with such a methodological comparison performed elsewhere^9^, and indicate that a detection dog can provide consistency when reliable assistants are unavailable.

Working with an established scat-detection dog group offers substantial benefits if one’s research budget allows for it. Becoming a dog handler requires extensive training, and the energy, intelligence, and temperament of these dogs demands considerable time and attention. The dog must be cared for, transported, and housed safely and responsibly during all phases of the research project, which is not always simple in cultures that lack familial human-canine relationships. Additionally, the handler’s responsibility does not end with the research project. If suitable permanent housing cannot be found for the detection dog in country, it is not merely negligent but an ethical failure to leave such a highly intelligent service animal behind. The established scat-detection dog groups perform exemplary work, not only because of their experience and expertise, but also because of their respect for their service animals. The commitment levied upon the researcher who chooses to become a dog handler or establish a local detection dog program cannot be overestimated. As discussed, the main obstacles to the expanded use of scat-detection dogs in conservation and molecular ecology have been the financial and logistical hurdles of hiring a detection dog team from the United States.

We have demonstrated that it is mutually advantageous for conservation biologists to work closely with a local police dog training base. First, our approach offers a savings of one to two orders of magnitude (tens to hundreds of thousands of dollars) when compared to other detection dog studies that have provided cost estimates^7^,^8^. The direct cost of our dog and his training was $2,351 (in 2015 dollars). Indirect costs of international dog transportation, quarantine, and foreign handler services were eliminated. Other essential costs (food, local transportation, housing, etc.) totaled less than $500. Secondly, working with local police can substantially improve project logistics over alternative approaches by facilitating travel, long- and short-term dog housing, and interaction with permit officials, field assistants, and other local actors. By collaborating as American and Chinese academics with the Chinese police, we were able to overcome linguistic, cultural, and bureaucratic issues that until now have made such a project unfeasible. Finally, working with police dog trainers builds local capacity in the host country. By establishing the viability of our approach, it is now possible for our group to continue long-term genetic sampling and monitoring of primates in Yunnan. Likewise, other researchers and conservation organizations can now feasibly employ a detection dog team in China by working with the Kunming Police Dog Training Base to train dogs to suit their needs. The widespread regional publicity generated by our project has brought further esteem to KPDTB, generating goodwill and affording them increased opportunities throughout China and Southeast Asia. Many countries have access to police dog trainers, and we strongly encourage the adoption of our practices where appropriate.

## Methods

### Ethics Statement

All methods were carried out in accordance with the *Principles for Ethical Treatment of Non-Human Primates* set forth by The American Society of Primatologists and the guidelines of the Institutional Animal Care and Use Committee (IACUC) of Washington University in St. Louis. All experimental protocols were approved by the Washington University in St. Louis Animal Studies Committee (A-3381-01 20090204). Permits to collect fecal samples were issued by the Yunnan Forestry Department (
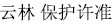
 55 [2009] 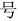).

### Study Sites

We conducted our field work in two forested regions of Yunnan: Wuliangshan National Nature Reserve and Yongde Daxueshan National Nature Reserve. Both reserves are sky island habitats, isolated from neighboring forested areas by continuous lowland agriculture. Wuliangshan National Nature Reserve is located in central Yunnan Province, between the Mekong (Lancang Jiang) and Black (Chuan He) Rivers in the upper reaches of the Wuliang Mountains (Fig. 2). The reserve covers 31, 313 ha in Jingdong, Nanjian, and Zhengyuan counties, with a length of 83 km and a variable width (~5–15 km)^34^. Yongde Daxueshan (15, 786 ha) is located in Yongde County, Yunnan, about 100 km west of Wuliangshan and near the Burmese border^35^. The forest structure of both sites is largely similar. Within the suitable primate habitat (~1,800–2,700 m above sea-level), the forest grades from semi-humid evergreen broadleaf forest through a sub-humid evergreen broadleaf zone, above which the forest grades into dwarf rhododendron forest. Inside the reserve, these sites contain a mixture of primary and secondary forest with a greater degree of the former at higher altitudes^36^,^37^. Four species of primates are known to be codistributed throughout Wuliangshan (*Nomascus concolor*, *Trachypithecus crepusculus*, *Macaca arctoides*, and *M. mulatta*)^38^ and Yongde Daxueshan (*N. concolor*, *T. crepusculus*, *M. arctoides*, and *Macaca assamensis, M. leonina*). In both mountains, small felids, deer, serows, gorals, flying squirrels, and songbirds are fairly common; bears, leopards, and raptors have also been reported in small numbers, but their present status is unknown.

Wuliangshan and Yongde Daxueshan are protected areas; however, human perturbations of the forest are commonplace, and include encroachment of grazing goats and cattle, small scale logging for house beams and firewood, and foraging for mushrooms. Previously forested areas outside the nature reserves have been almost exhaustively cleared for plantations (e.g. tea, walnuts, corn). Hunting is prohibited, but hunting pressure remains, predominantly for medicinal and commercial purposes. Steel offset-jaw leg traps (“bear traps”), rope snare spring-noose traps, and small caliber bullet shells are present in both reserves, but are more common in Yongde Daxueshan. The Jingdong Management Bureau of Wuliangshan National Nature Reserve has halted primate hunting in Wuliangshan thanks to aggressive conservation efforts, but conservation in Yongde Daxueshan remains challenging, and primates are occasionally discovered deceased.

### Study Animals

**Figure 1:**
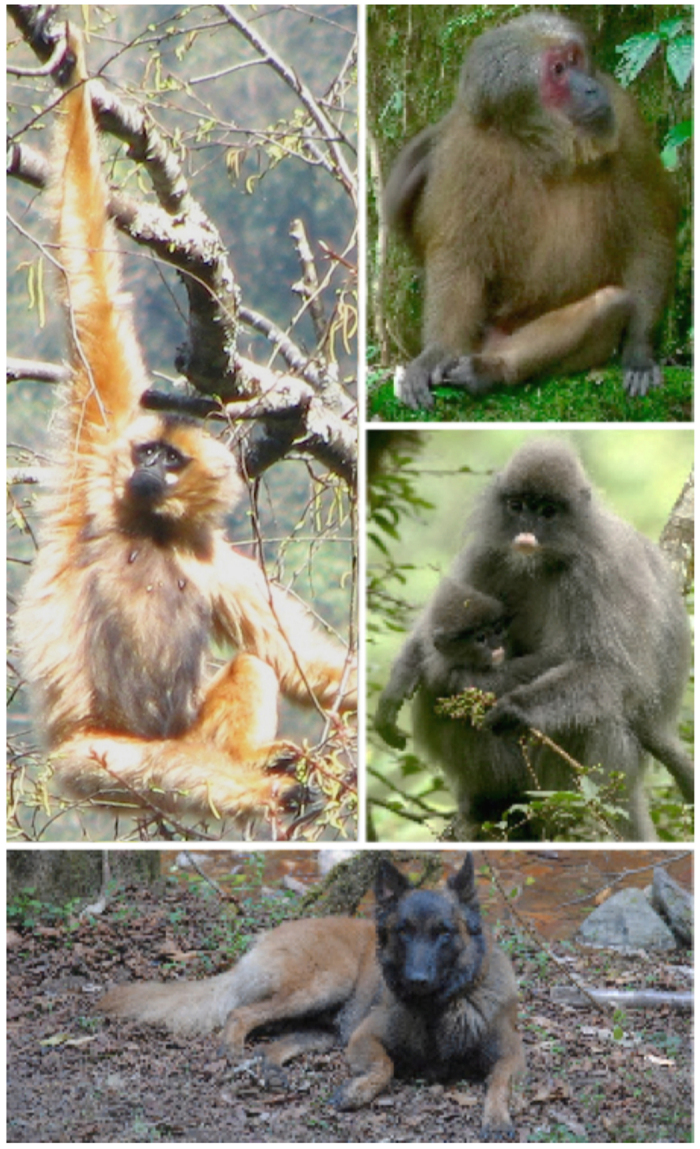
Clockwise from top left: western black crested gibbon (*Nomascus concolor*), stump-tailed macaque (*Macaca arctoides*), Indochinese gray langur (*Trachypithecus crepusculus*), and Pinkerton, the scat-detection dog. Photos courtesy of Peng-Fei Fan, and Joseph Orkin.

**Figure 2:**
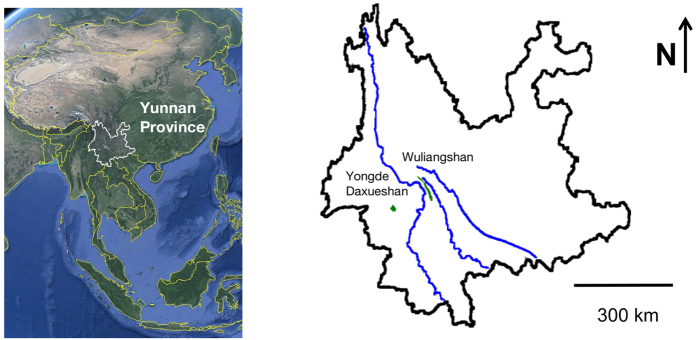
Left: Yunnan Province, China. Right: Study sites in Yunnan from which primate fecal samples were collected. Nature reserves are indicated with green polygons. The Mekong, Black, and Red Rivers are drawn in blue from west to east, respectively. Figures were generated with Esri ArcMap 10.2 (https://www.arcgis.com) and Google Earth 7.1 (https://www.google.com/earth/). Map data: Google, Landsat.

**Figure 3:**
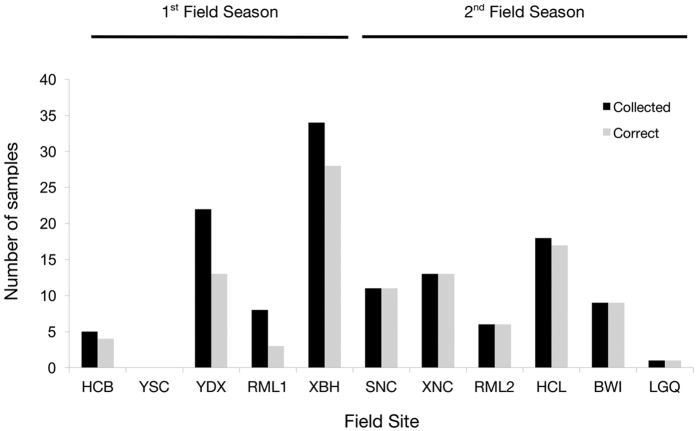
Primate scat samples identified by dog team over time (HCB → BWI). Error decreases substantially in the second field season. HCB = Huangcaoba, YSC = Yenshancun, YDX = Yongde Daxueshan, RML = Raomalu, XBH = Xiaobahe, SNC = Shaniucun, HCL = Huangcaoling, BWI = Bangwai, LGQ = Langanqing.

Gibbons, langurs, and macaques occupy different ecological niches (Fig. 1). Gibbons are near obligate arborealists, relying on brachiation for locomotion and rarely descending to the forest floor^30^,^39^, whereas langurs and macaques are quadrupedal primates occupying a mixed terrestrial/arboreal niche. The gibbons of central Yunnan have a more variable diet than many gibbons, consuming an average of 50% leaves and buds, 43% fruit, and 5% flowers from 83 plant species^33^,^40^,^41^. Indochinese gray langurs in Wuliangshan have been observed to consume a diverse, leaf-heavy diet (148 plant species; 54% leaves and buds, 32% fruit, 6% flowers, and 6% soil)^42^. Their high level of folivory is also evidenced by morphological adaptations for foregut fermentation^32^. Macaques have a versatile diet, variably composed by different species at different sites^31^, but the particular diets of the macaques at our study sites have not been investigated thoroughly.

Primate populations in both Wuliangshan and Yongde Daxueshan are small and threatened. 435 individual (87 groups) western black crested gibbons are estimated to remain in Wuliangshan^43^, and a 2011 survey identified only 11 individuals—three groups and one solitary male—in Yongde Daxueshan. 1,960 individual (43 groups) Indochinese gray langurs were estimated to remain in the Wuliang Mountains (Jingdong County) in 2013^38^. Reliable census data are not available for the langurs in Yongde Daxueshan and the macaques in either reserve, but forest rangers and officials have seen both in recent years and believe them to be more abundant than gibbons.

### Detection Dog Training

Over the course of three months during the summer/autumn of 2010, the Kunming Police Dog Training Base of the Chinese Ministry of Security (KPDTB) trained a Belgian Malinois, named “Pinkerton”, to locate and identify scat from three species of primates (*N. concolor*, *T. crepusculus*, and *M. arctoides*). The KPDTB commonly trains Belgian Malinois for scent work, because of their potential for high levels of excitability, durability, adaptability, strong sense of smell, and hunting instincts. Prior to Pinkerton’s training, we collected scat for our species of interest from the Kunming Zoo with gloved hands to avoid scent contamination, deposited it into 50 ml plastic tubes, and desiccated it with an electric fan for preservation. While we were able to collect fresh scats from *M. arctoides* (four individuals) and *T. crepusculus* (two individuals), *N. concolor* and other macaques were not available. We collected feces from the closely related white-cheeked gibbon, *N. leucogenys* (4 individuals) instead. We then covered the purchase price of Pinkerton from the KPDTB and supplied the head trainer with fecal samples for the three primate species of interest. Pinkerton’s training began at the age of eight months; at this age, dogs can quickly learn and remember new skills. Pinkerton was trained to signal that scat was deposited by one of the three target species by placing his paws on either side of the scat and lying down beside it; he was not trained to differentiate among scats from the three target species. (See Supplemental Methods for detailed training protocols).

Once Pinkerton could reliably locate and identify primate scat, the trainer spent one month in Kunming (October 2010) habituating the field handler (J.D.O) to the dog and teaching him the search commands, visual cues, and other practices necessary to work with Pinkerton in the forest. During fieldwork, the handler practiced these techniques with Pinkerton at least twice per week during downtime in the forest or while waiting for transportation to field sites.

### Field Surveys

During the dry seasons (October–May) of 2010/11 and 2011/12, we visited nine sites in Wuliangshan known or presumed to be inhabited by our species of interest (Bangwai, Huangcaoba, Huangcaoling, Langanqing, Raomalu, Shaniucun, Xiaobahe, Xincun, and Yenshancun) for one to five weeks each. We camped in the forest within gibbon home ranges when the risk of forest fire was low; otherwise, we slept in the homes of our local assistants. At each site in the Wuliang Mountains, we hired one to four local field assistants, depending on their availability and knowledge of the forest. Although it was not always possible to work with a consistent number of field assistants, we split opportunistically into two search groups—a human-dog team and a human-only team—at Bangwai, Huangcaoba, Raomalu, Shaniucun, and Xincun. In such cases, two field assistants would collect fecal samples independently of the two-person human-dog team.

Fieldwork in Yongde Daxueshan occurred during a gibbon population census led by The Kunming Institute of Zoology (KIZ) and Flora and Fauna International (December 2010–January 2011). During this four-week survey, the survey team stopped at six unnamed listening posts in Yongde Daxueshan for two to four days each. The human-dog team was only able to search when the survey team stopped.

### Search Methods

We prioritized the search for gibbons, but we trained our dog to detect primate scat broadly so that we could collect samples from sympatric species with different diets and behaviors in a single effort. In mountainous habitats, gibbons are best located by climbing to high-elevation listening posts before dawn and waiting to identify the geographic origin of their morning songs^36^,^44^. When multiple field assistants were available, we occupied separate listening posts in an attempt to flank a gibbon group’s calling site. When we heard gibbons, we walked promptly to the presumed area of the calling tree (usually 1–2 km away). When gibbons did not call, we walked to locations where our assistants had seen primates in the past (usually small river bottoms, cliffs, and feeding trees). When contacting primates, we never approached them with the dog to reduce the risk of disease transmission. Because hunting dogs and cattle occasionally enter these forests, any potential disease risk from Pinkerton would not have constituted a novel threat.

Upon reaching a search area, the handler commanded Pinkerton to take the lead and search for primate feces along a path of the dog’s choosing. The handler followed five meters behind the dog with assistants five meters behind him. Because detection dogs are motivated by human affection, walking at some distance behind Pinkerton minimized distractions. When Pinkerton identified target feces, the handler rewarded him with a tennis ball and collected the sample. When a human team member identified a sample in disagreement with the dog it was collected and coded as human-identified. In cases where multiple field assistants were available we opportunistically separated into a dog-human team and a human-only team to broaden our search for primate feces.

### Fecal Sample Collection

Fecal samples for genetic analysis were collected following the two-step ethanol and silica method^45^. All fecal samples were collected with gloved hands and sticks from the forest floor to minimize human contamination. In order to avoid cross-contamination of fecal samples from different individuals, fragmentary samples were placed in separate collection tubes whenever it was not obvious that the scats originated from the same bolus. Samples were kept at room temperature for one week to two months until they could be frozen at −20 °C in Jingdong City, Yunnan, then frozen at −80 °C at KIZ.

### Laboratory Analysis

Genetic work was conducted at KIZ. DNA was extracted using the 2CTAB/PCI method^46^ under a UV sterilized fume hood with unidirectional airflow. We wore facemasks and lab coats at all times during extraction. To determine species or genus of origin, we attempted to PCR amplify taxonomically informative genetic markers for all collected fecal samples. Although DNA barcoding is typically done with the CO1 locus^47^, we sought to facilitate future phylogenetic and conservation genetic research on these endangered primates by first amplifying mitochondrial d-loop sequences where possible. Our approach of amplifying a series of taxonomically informative markers until the genus (or species) of origin could be identified also minimized lab costs. Given the presumption that most fecal samples would have been from *Nomascus concolor*, we began by using primers that we designed to amplify ~600 bp of the d-loop in *Nomascus* (Table S1). For samples that did not amplify or provided unintelligible sequencing reads, we then used a d-loop primer from which we had successfully amplified both *Trachypithecus* and *Macaca* mtDNA (Table S1). For the remaining samples, it was possible that they were 1) too degraded to amplify a ~600 bp mtDNA region, and thus of limited future use; 2) from another organism; or 3) a combination of both factors. To resolve the identity of these remaining samples, we attempted to amplify a vertebrate-specific 220 bp CO1 mini-barcode locus^48^ that would yield taxonomically informative information, albeit of limited future use.

Each PCR was performed with negative controls in a 25 μl reaction containing 2.5 μl of 10x buffer, 1 μl dNTP, 1 μl BSA, 1 μl of each primer, 0.5 μl of DNA, 0.125 μl of TaKaRa Hot Start taq polymerase, and 17.875 μl of millipure water. Thermocycling conditions were as follows: an initial denaturation of 3 min at 94 °C followed by 10 cycles of 45 s at 94 °C, 1 min at 55 °C with a reduction of 0.5 °C each cycle, and 1 min at 72 °C; this was followed by 25 cycles of 45 s at 94 °C, 1 min at 50 °C, and 1 min at 72 °C and a final annealing stage of 10 min at 72 °C. For samples that did not amplify, a 25 μl secondary PCR was amplified using a 1:10 dilution of the initial PCR product in place of template DNA. PCR purification and Sanger sequencing were conducted at The Beijing Genomics Institute on an ABI 3730xl cycle sequencer.

### Taxonomic Assignment

Geneious R7^49^ was used to trim reads of low quality bases and assemble contigs from the bidirectional reads from each sequenced PCR amplification. Each contig was aligned to the NCBI database using nBLAST with default parameters. Tabular nBLAST output was downloaded to Geneious R7, and the genus of origin for each sample was determined identifying the nBLAST alignment first with the lowest E value, and second by the Geneious “Grade,” which is the weighted average of the E score, pairwise identity, and coverage, if multiple alignments had equivalent E values. Potential human- or cross-contamination of the samples was screened by the presence of multiple traces in sequencing chromatograms. All unique sequences have been submitted to GenBank with accession codes available in Table S2.

### Assessment of Detection Dog Success Rate

All collected samples were coded first by whether they yielded DNA that amplified with our primer set, and secondly whether we could taxonomically identify them to one of our target animals. For both the dog-human team and the human-only team, detection accuracy was defined as the proportion of samples from which target animal DNA could be identified relative to the total number of samples that yielded amplifiable DNA. Because of the hierarchical structure of our sampling methods (two mountains, each with multiple sites), we applied a linear mixed effects model to discern if the dog-team had a significantly greater accuracy and collection rates than the human-only teams. Sampling sites were nested within mountains as random effects with sample collector (dog or human) as a fixed effect. Models were generated in R with the package nlme. P-values were adjusted for significance using Benjamin-Hochberg false discovery rates.

## Additional Information

**How to cite this article**: Orkin, J. D. *et al.* Cost-effective scat-detection dogs: unleashing a powerful new tool for international mammalian conservation biology. *Sci. Rep.* **6**, 34758; doi: 10.1038/srep34758 (2016).

## Supplementary Material

Supplementary Information

## Figures and Tables

**Table 1:** Fecal samples collected from primates in Yunnan *with* identification by the dog team.

Species	Putative scats collected	Successful DNA extraction	DNA confirmed scats	Dog accuracy	Days surveyed
Western black crested gibbon (*Nomascus concolor*)	127	112 (89%)	61	92%	180
Indochinese gray langur (*Trachypithecus crepusculus*)			9		
Macaque (*Macaca* sp.)			33		
Total			103		

**Table 2:** Fecal samples collected from primates in Yunnan *without* identification by the dog team.

Species	Putative scats collected	Successful DNA extraction	DNA confirmed scats	Dog accuracy	Days surveyed
Western black crested gibbon(*Nomascus concolor*)	75	45 (60%)	10	76%	180
Indochinese gray langur (*Trachypithecus crepusculus*)			12		
Macaque (*Macaca* sp.)			12		
Total			34		

**Table 3:** ANOVA results from hierarchical mixed effect linear models used to determine the effectiveness of the dog-human team against the human-only team.

Model	numDF	denDF	F-value	p-value	p-adjusted
1) Correct ~ Collected + Team | Mountain/Site					
Intercept	1	12	182.07	<0.001	0.003
Collected	1	12	304.64	<0.001	**0.003**
Team	1	12	6.94	0.022	**0.034**
2) Correct ~ Collected/DNA + Team | Mountain/Site					
Intercept	1	12	196.32	<0.001	0.003
Collected	1	11	318.87	<0.001	**0.003**
Team	1	1	6.90	0.024	**0.034**
Collected:DNA	1	11	1.89	0.196	0.238
3) Incorrect ~ Collected/DNA + Team | Mountain/Site					
Intercept	1	12	5.00	0.045	0.059
Collected	1	11	0.00	0.990	0.990
Team	1	11	0.11	0.748	0.795
Collected:DNA	1	11	0.97	0.324	0.388
4) Unidentifiable ~ Collected + Team | Mountain/Site					
Intercept	1	12	45.45	<0.001	0.003
Collected	1	12	25.08	0.003	**0.006**
Team	1	12	13.19	0.003	**0.006**
5) Useless ~ Collected + Team | Mountain/Site					
Intercept	1	12	40.98	<0.001	0.003
Collected	1	12	9.52	0.009	**0.017**
Team	1	12	6.94	0.022	**0.034**

Each model accounts for the nested sampling design, wherein samples were collected at multiple sites within two mountain ranges. Model 1: effect of team membership on the number of DNA confirmed primate samples, accounting for the number of collected samples. Model 2: effect of team membership on the number of DNA confirmed primate samples, accounting for the number of collected samples from which DNA could be amplified. Model 3: effect of team membership on the number of DNA confirmed non-primate samples, accounting for the number of collected samples from which DNA could be amplified. Model 4: effect of team membership on the number of collected samples from which no DNA could be amplified, accounting for the number of collected samples. Model 5: effect of team membership on the combined number of DNA confirmed non-primate samples and samples from which no DNA could be amplified, accounting for the number of collected samples. Significant (FDR adjusted p < 0.05) predictors are in bold.
