# Supplementary material for: Cost-effective scat-detection dogs: unleashing a powerful new tool for international mammalian conservation biology
Source: Sci Rep. 2016 Oct 10;6:34758. doi: 10.1038/srep34758 (PMC5056371; doi:10.1038/srep34758)
Supplement: Supplementary Information [file srep34758-s1.pdf]

Title: Cost-effective scat-detection dogs: unleashing a powerful new tool for international mammalian conservation biology

Authors: Joseph D. Orkin[1,2], Yuming Yang[3], ChunyanYang[1], Douglas W. Yu[1,4], Xuelong Jiang[1*]

SUPPLEMENTARY INFORMATION

SUPPLEMENTAL METHODS

Following basic obedience skills, scat-detection training ensued by incrementally increasing the complexity of search-related tasks over time to receive a reward. We began by heightening Pinkerton's possessive instinct; this was done by repeatedly teasing him with a tennis ball or rolled towel. Once a desire for these objects was formed in Pinkerton's mind, we began placing these objects in close proximity to the zoo scats. Next, scats were placed in easy to locate places; when Pinkerton expressed interest in them, he was rewarded with the aforementioned toys or food treats. Pinkerton was then instructed to lie down with his paws on either side of a scat after he had smelled it in order to receive his reward.

Once Pinkerton achieved a basic level of detection ability—the capacity to identify a target species scat by lying beside it to receive a reward—we increased the difficulty of finding target scat. We began with a "Multiple Block Test," in which we hid Pinkerton's toy or primate scat underneath one of several sequentially aligned cinder blocks (Figure S1). This test allows for a small amount of uncertainty: the dog observes the handler placing scat in one of four places within a small, circumscribed area, but it does not know which. By hiding the scat underneath a cinderblock, the dog cannot rely on visual cues and must identify it by smell alone. Only when Pinkerton had lain down with paws beside the correct cinderblock was he rewarded for proper identification. Secondly, we trained Pinkerton with a "Large Area Test," in which we hid one to five scats randomly in an open field without the dog observing the locations. Pinkerton would be

given a command to commence searching, while the handler trailed several meters behind him, remaining within sight of the dog at all times. The large area test was then increased in complexity by moving into more complex terrain (e.g. forest) and only rewarding the dog upon his identification of multiple target scats.

SUPPLEMENTAL TABLES

Table S1: Primers used for genetic identification of fecal samples.

Taxon	Locus	Length (bp)	Primer name	Primer sequence
Nomascus	d-loop	600	GDL-L1 F	CGAAAACAAAATACTCAAATGAACCT
			GDL-H2 R	GGTGATCCATCGTGATGTCTTATT
Trachypithecus and *Macaca*	d-loop	749	TP-DL-L1 F	ACTACCATTAGCCTCCCTAATCG
			TP-DL-H2 R	GATATTGATTTCACGGAGGATGG
Vertebrates	CO1	220	CO1-220-F	TAYTACGTTGTAGCCCACTTCCAC
			CO1-220-R	GGGTAGTCCGAGTAWCGTCG

Table S2:

Genbank Accession	Organism	Isolate	Haplotype
TBD	*Nomascus concolor*	South China Wuliang Mountain	BWI01
TBD	*Nomascus concolor*	South China Wuliang Mountain	BWI03
TBD	*Nomascus concolor*	South China Wuliang Mountain	BWI05
TBD	*Nomascus concolor*	South China Yongde Daxue Mountain	YDX201
TBD	*Nomascus concolor*	South China Yongde Daxue Mountain	YDX408
TBD	*Nomascus concolor*	South China Wuliang Mountain	XNC20
TBD	*Nomascus concolor*	South China Wuliang Mountain	XNC17
TBD	*Nomascus concolor*	South China Wuliang Mountain	XNC16
TBD	*Nomascus concolor*	South China Wuliang Mountain	XBH13
TBD	*Nomascus concolor*	South China Wuliang Mountain	XBH12
TBD	*Nomascus concolor*	South China Wuliang Mountain	XBH10
TBD	*Nomascus concolor*	South China Wuliang Mountain	XBH202
TBD	*Nomascus concolor*	South China Wuliang Mountain	XBH203

TBD	*Nomascus concolor*	South China Wuliang Mountain	XBH07
TBD	*Nomascus concolor*	South China Wuliang Mountain	XBH06
TBD	*Nomascus concolor*	South China Wuliang Mountain	SNC14
TBD	*Nomascus concolor*	South China Wuliang Mountain	SNC11
TBD	*Nomascus concolor*	South China Wuliang Mountain	SNC10
TBD	*Nomascus concolor*	South China Wuliang Mountain	RML27
TBD	*Nomascus concolor*	South China Wuliang Mountain	HCL14
TBD	*Nomascus concolor*	South China Wuliang Mountain	HCL12
TBD	*Nomascus concolor*	South China Wuliang Mountain	HCL08
TBD	*Nomascus concolor*	South China Wuliang Mountain	HCL07
TBD	*Nomascus concolor*	South China Wuliang Mountain	HCL06
TBD	*Nomascus concolor*	South China Wuliang Mountain	HCL04
TBD	*Nomascus concolor*	South China Wuliang Mountain	HCL01
TBD	*Nomascus concolor*	South China Wuliang Mountain	HCB22
TBD	*Nomascus concolor*	South China Wuliang Mountain	HCB03
TBD	*Trachypithecus crepusculus*	South China Wuliang Mountain	HCB13
TBD	*Trachypithecus crepusculus*	South China Wuliang Mountain	HCB14
TBD	*Trachypithecus crepusculus*	South China Wuliang Mountain	HCB17
TBD	*Trachypithecus crepusculus*	South China Wuliang Mountain	HCB18
TBD	*Trachypithecus crepusculus*	South China Wuliang Mountain	RML23

TBD	*Macaca arctoides*	South China Wuliang Mountain	XBH16
TBD	*Macaca arctoides*	South China Wuliang Mountain	XNC09
TBD	*Macaca arctoides*	South China Yongde Daxue Mountain	YDX401
TBD	*Macaca sp.*	South China Wuliang Mountain	HCL19
TBD	*Macaca sp.*	South China Yongde Daxue Mountain	YDX208
TBD	*Macaca sp.*	South China Yongde Daxue Mountain	YDX209
TBD	*Macaca sp.*	South China Yongde Daxue Mountain	YDX212
TBD	*Macaca sp.*	South China Yongde Daxue Mountain	YDX602
TBD	*Macaca sp.*	South China Yongde Daxue Mountain	YDX604

SUPPLEMENTAL FIGURES

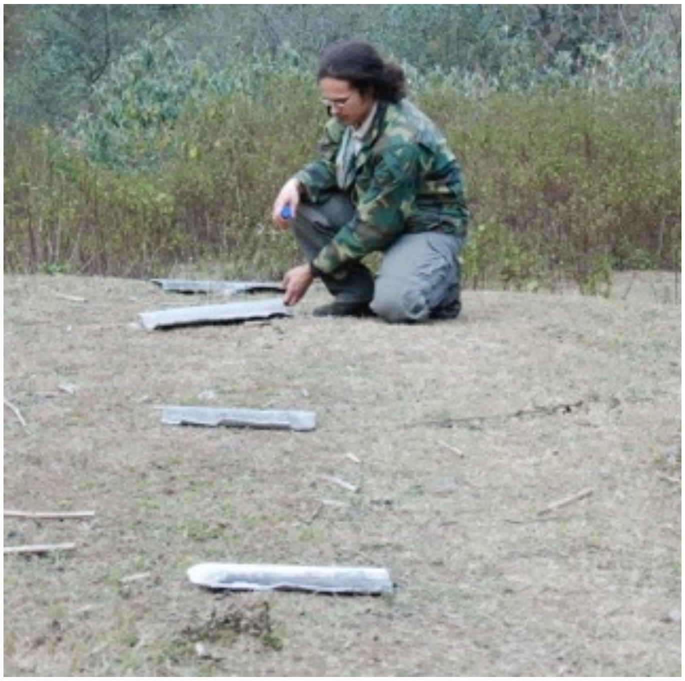

Figure S1: Multiple block test: Handler hiding *Nomascus* scat underneath the third in a sequence of tiles. The dog is then released to locate the scat. This test is easily modified for field conditions by substituting any available cover for the tiles or cinderblocks.
